# Supplementary material for: Structure of the human dopamine transporter and mechanisms of inhibition
Source: Nature. 2024 Aug 7;632(8025):672–7. doi: 10.1038/s41586-024-07739-9 (PMC11324517; doi:10.1038/s41586-024-07739-9)
Supplement: Supplementary file 1 — This file contains Supplementary Table 1 and Supplementary Figs. 1–9. [file 41586_2024_7739_MOESM1_ESM.pdf]

nature portfolio

https://doi.org/10.1038/s41586-024-07739-9

Supplementary information

Structure of the human dopamine transporter and mechanisms of inhibition

In the format provided by the authors and unedited

Supplementary Information

Structure of the human dopamine transporter and mechanisms of inhibition

Dushyant Kumar Srivastava[1], Vikas Navratna[1#], Dilip K. Tosh[2], Audrey Chinn[1], Md Fulbabu Sk[3,4,5], Emad Tajkhorshid[3,4,5], Kenneth A. Jacobson[2*] and Eric Gouaux[1,6*]

[1]Vollum Institute, Oregon Health and Science University, 3232 SW Research Drive, Portland, OR, USA.

[2]Molecular Recognition Section, Laboratory of Bioorganic Chemistry, National Institute of Diabetes and Digestive and Kidney Diseases, National Institutes of Health, 9000 Rockville Pike, Bethesda, MD, USA.

[3]Theoretical and Computational Biophysics Group, NIH Center for Macromolecular Modeling and Bioinformatics, Beckman Institute for Advanced Science and Technology, University of Illinois at Urbana-Champaign, Urbana, IL, USA

[4]Department of Biochemistry University of Illinois at Urbana-Champaign, Urbana, IL, USA

[5]Center for Biophysics and Quantitative Biology, University of Illinois at Urbana-Champaign, Urbana, IL, USA

[6]Howard Hughes Medical Institute, Oregon Health and Science University, 3232 SW Research Drive, Portland, OR, USA.

[*]Correspondence to: Kenneth A. Jacobson, kennethj@niddk.nih.gov or

Eric Gouaux, gouauxe@ohsu.edu

[#] Present address: Life Sciences Institute, University of Michigan, Ann Arbor MI, USA

Supplementary Table 1. Percentage of stacking of MRS7292 and W84 of ∆-hDAT.

Simulation	Total Frames	Total Stacked Frames	% of Stacked Frames
Replica 1	100,000	99,957	99.96
Replica 2	100,000	99,495	99.49
Replica 3	100,000	99,465	99.46
Replica 4	100,000	99,305	99.30
Replica 5	100,000	99,761	99.76

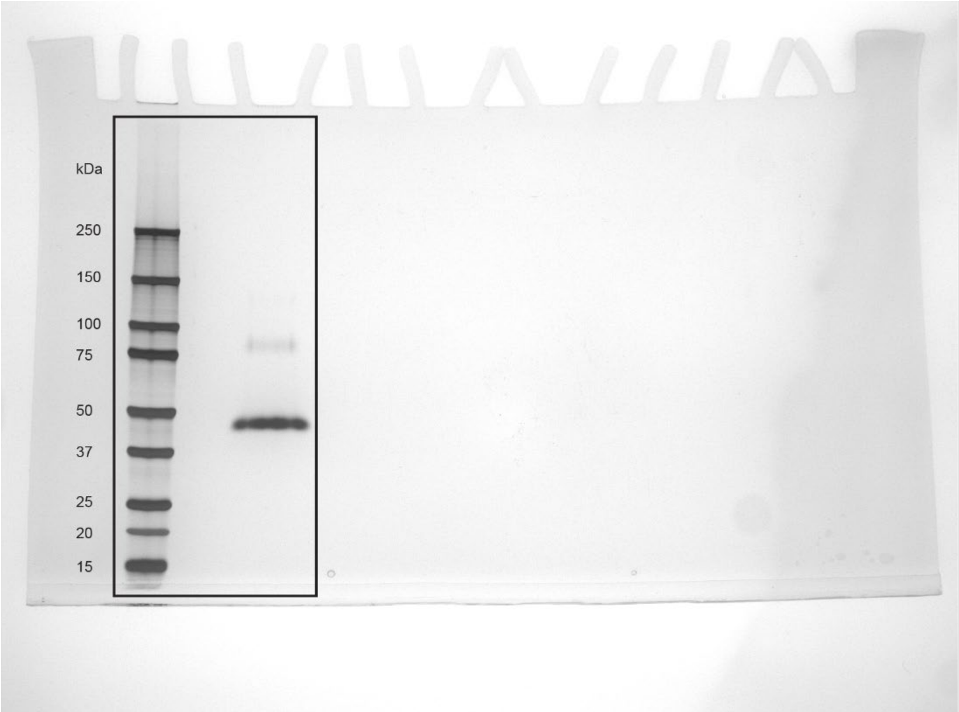

Supplementary Figure 1. Source data for silver-stained gel (see Extended Data Fig. 1b). The black box indicates how the image was cropped.

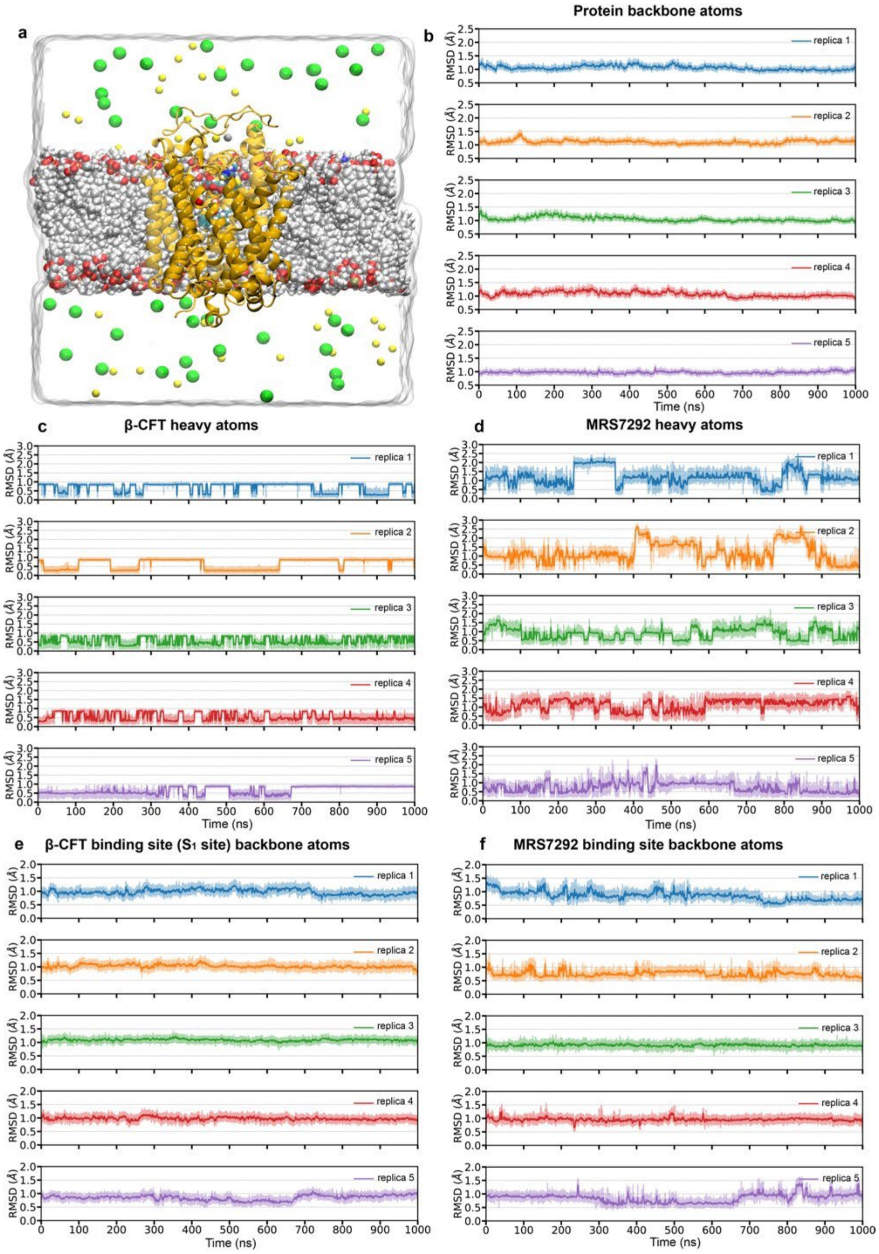

Supplementary Figure 2. (a) Initial setup for the five simulation replicas. Δ-hDAT (orange) is in the outward-facing open (OF) state bound with the ligands (MRS7292 and β-CFT; cyan space-filling). The lipids are shown as silver space-filling spheres, water is in transparent surface representation, and Cl^- and Na^+ ions are in green and yellow spheres, respectively. (b) Time evolution of Δ-hDAT backbone atoms (TM helices) RMSD values with respect to their initial position in the cryo-EM model is shown for the five simulation replicas. (c-d) Heavy-atom RMSD values of β-CFT and MRS7292 with respect to their initial positions in the cryo-EM model are shown for the five simulation replicas (trace colors are the same as those used for the protein RMSD in (b)). MRS7292 moderately fluctuates throughout the simulations (RMSD value ranging from 0.7 to 1.20 Å), while β-CFT remains fully stable in the S_1 site throughout the simulations (RMSD ≈ 0.62 Å). (e-f) Time series of RMSD values of the binding pocket (S_1 or central site and S_2 or MRS allosteric site) backbone atoms with respect to their initial position in the cryo-EM model. Both pockets remain stable throughout the simulations (RMSD ≈ 1.0 Å).

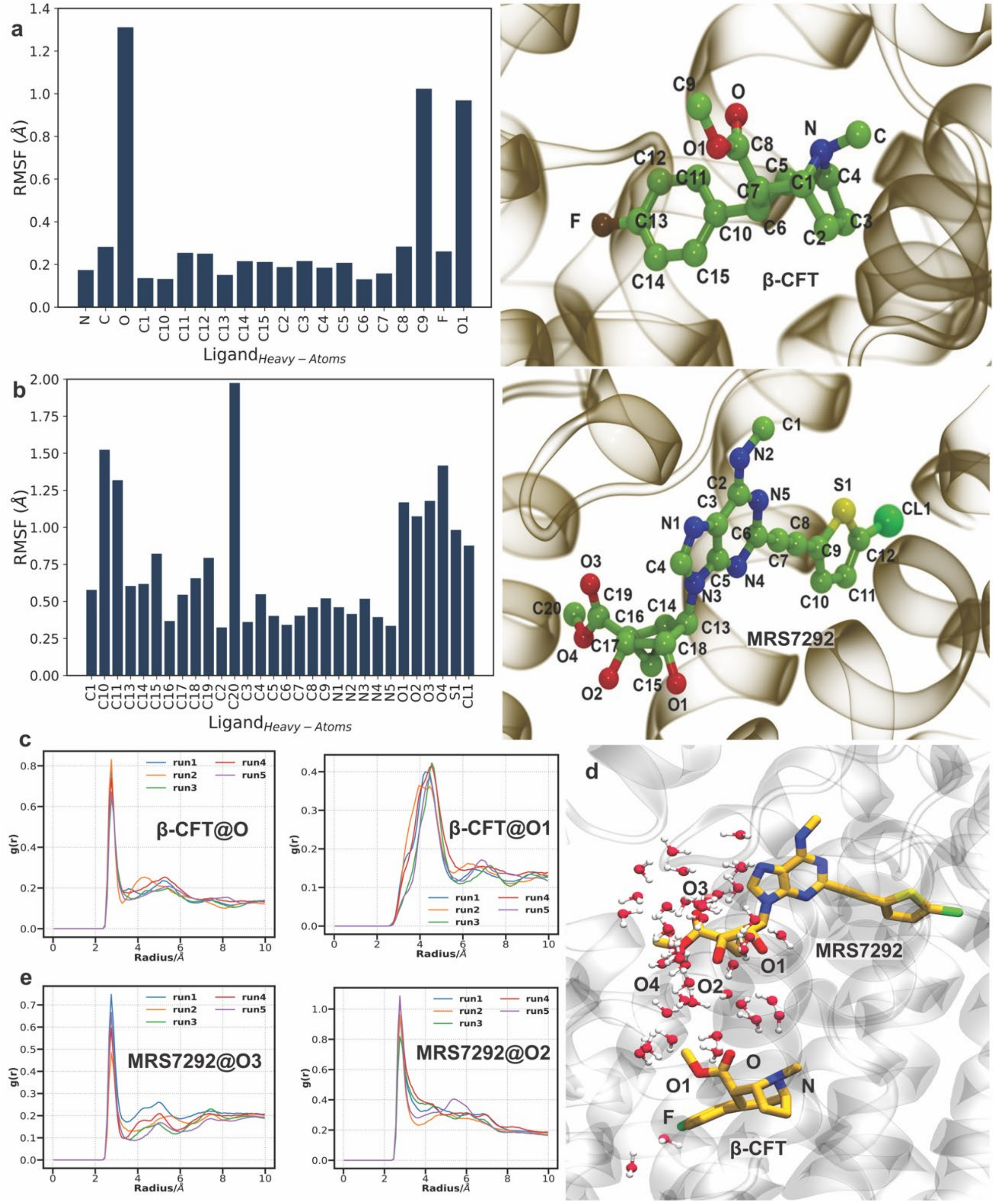

Supplementary Figure 3. (a-b) The atomic root mean-square fluctuation (RMSF) of β-CFT and MRS7292 from the last 500 ns of the five simulation replicas. RMSF values are calculated with respect to their initial positions in the cryo-EM structure. (c-d) The radial distribution functions (RDFs) of the oxygen (O) atoms of water around the ligands (ligand-O distance) calculated for each simulation replica. Here, we considered only O, N, Cl and F atoms of the MRS7292 and β-CFT molecules as anchors, and the RDF is calculated from the histogram of the number of waters as a function of distance, normalized by the expected number of water molecules at that distance (water density 1.0 g/mL).

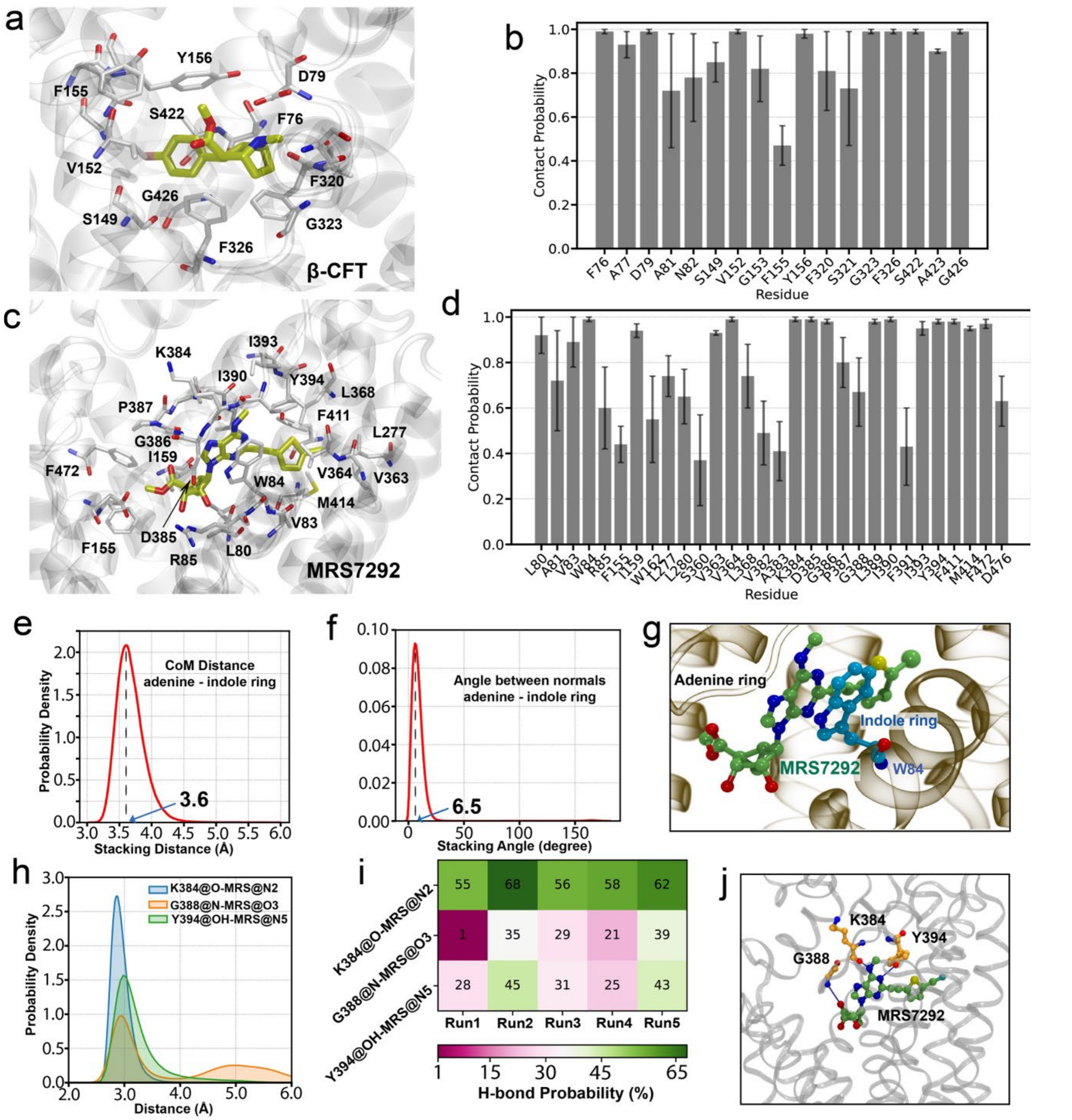

Supplementary Figure 4. Summary of MD simulation of ∆-hDAT dynamics embedded into membrane lipid. (a and c) Close-up views of β-CFT (S_1 site) and MRS7292 binding (S_2 site) are shown in boxes. Snapshots of the strongest interacting residues and representative binding modes of β-CFT and MRS7292 binding are highlighted. Both β-CFT and MRS7292 are depicted in licorice green representation. (b and d) Contact probability of ∆-hDAT residues with β-CFT and MRS7292 molecules. Contact probability is defined as ∆-hDAT residues (heavy atoms) within 4 Å of the ligand (heavy atoms) of interest. The total number of contact frames was tallied and normalized. The mean and standard deviation (SD) is derived from n=5 replicas. (e-g) Highlighting an essential interaction between ∆-hDAT (indole ring of W84 shown in blue) and MRS7292 (adenine ring, drawn in green). This includes the probability of their center of mass (COM) distance (stacking distance) and the angle between their normal vectors (stacking angle). The COM distance distribution peaks at 3.6 Å and an angle of 6.5 degrees between their normal vectors, corresponding to stabilization of MRS7292 by W84 in the S_2 site. (h-i) The distribution of selected interactions (as shown in (i); hydrogen bond distance) and their hydrogen bond probability, resulting from

the 5 µs MD simulation. (j) MRS7292 heavy atoms' distance distributions of K384, G388, and Y394 are presented as probability density functions (PDFs) in blue, orange, and green, respectively.

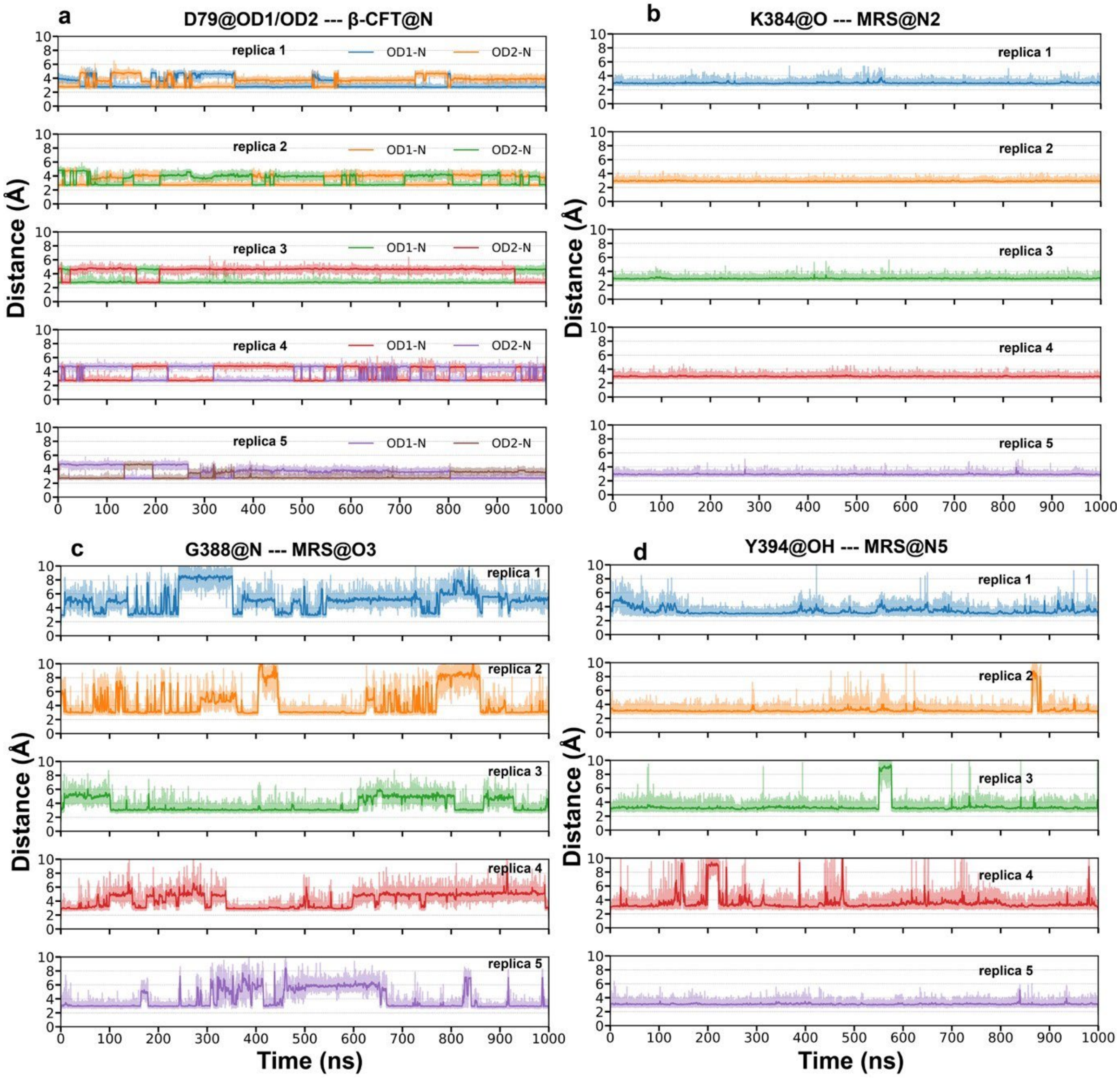

Supplementary Figure 5. Time evolution of hydrogen bond distances between (a) Δ-hDAT: D79 and β-CFT, and (b-d) Δ-hDAT residues K384, G388, and Y394 and MRS7292. The heavy atoms used for the calculations are mentioned above each panel.

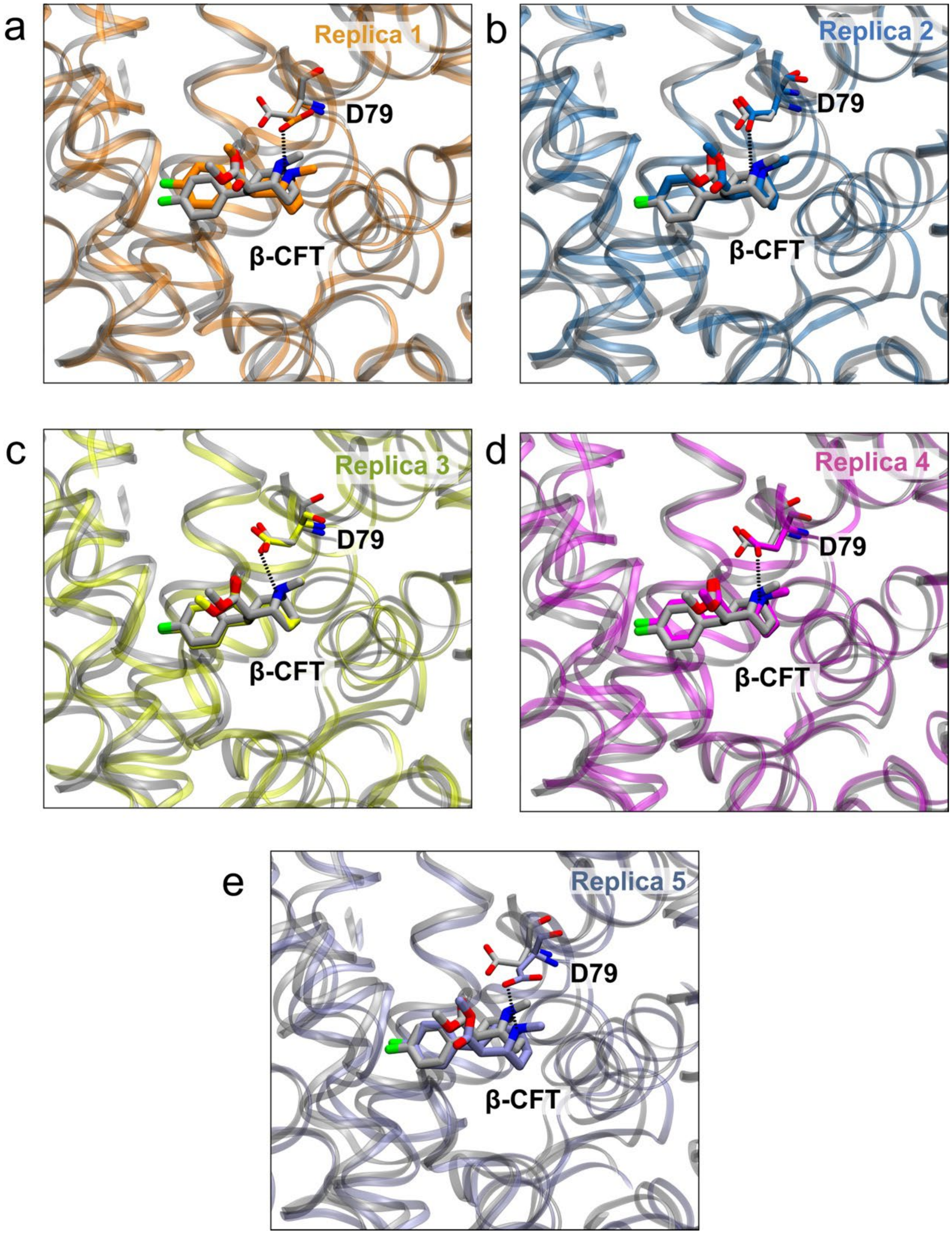

Supplementary Figure 6. Comparing the cryo-EM ligand pose and those in the five simulation replicas of β-CFT. (a-e) The superimposed close-up view of the S_1 site and β-CFT in the S_1 pocket. The cryo-EM model is shown in silver in each panel, and the final conformations of simulation replicas 1-5, are shown in orange, blue, light green, magenta, and lavender, respectively. One important hydrogen bond between Δ-hDAT:D79 and β-CFT@N is shown with dashed lines. Δ-hDAT residues are depicted in stick representation.

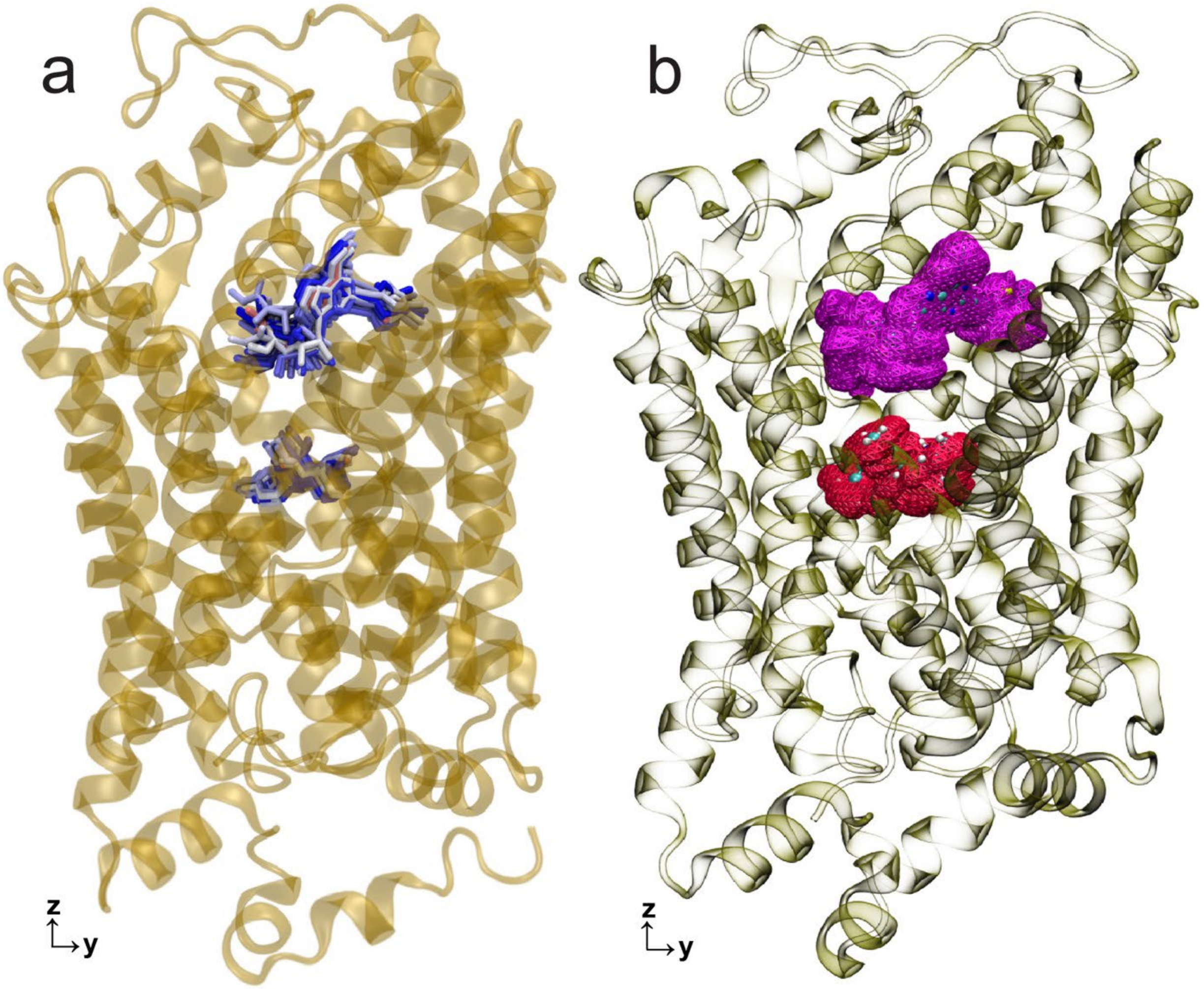

Supplementary Figure 7. β-CFT and MRS7292 sampling space within the ∆-hDAT central and allosteric sites. (a) Superimposed snapshots of the ligands (β-CFT and MRS7292) at 100 ns intervals (from all simulation replicas). The color from blue to red represents the initial to final snapshots. (b) Spatial (in-silico) density of the ligands obtained from the last 500 ns (from all the simulation replicas), with purple and red meshes corresponding to MRS7292 and β-CFT molecules, respectively. Each density mesh is shown at an isovalue of 0.3.

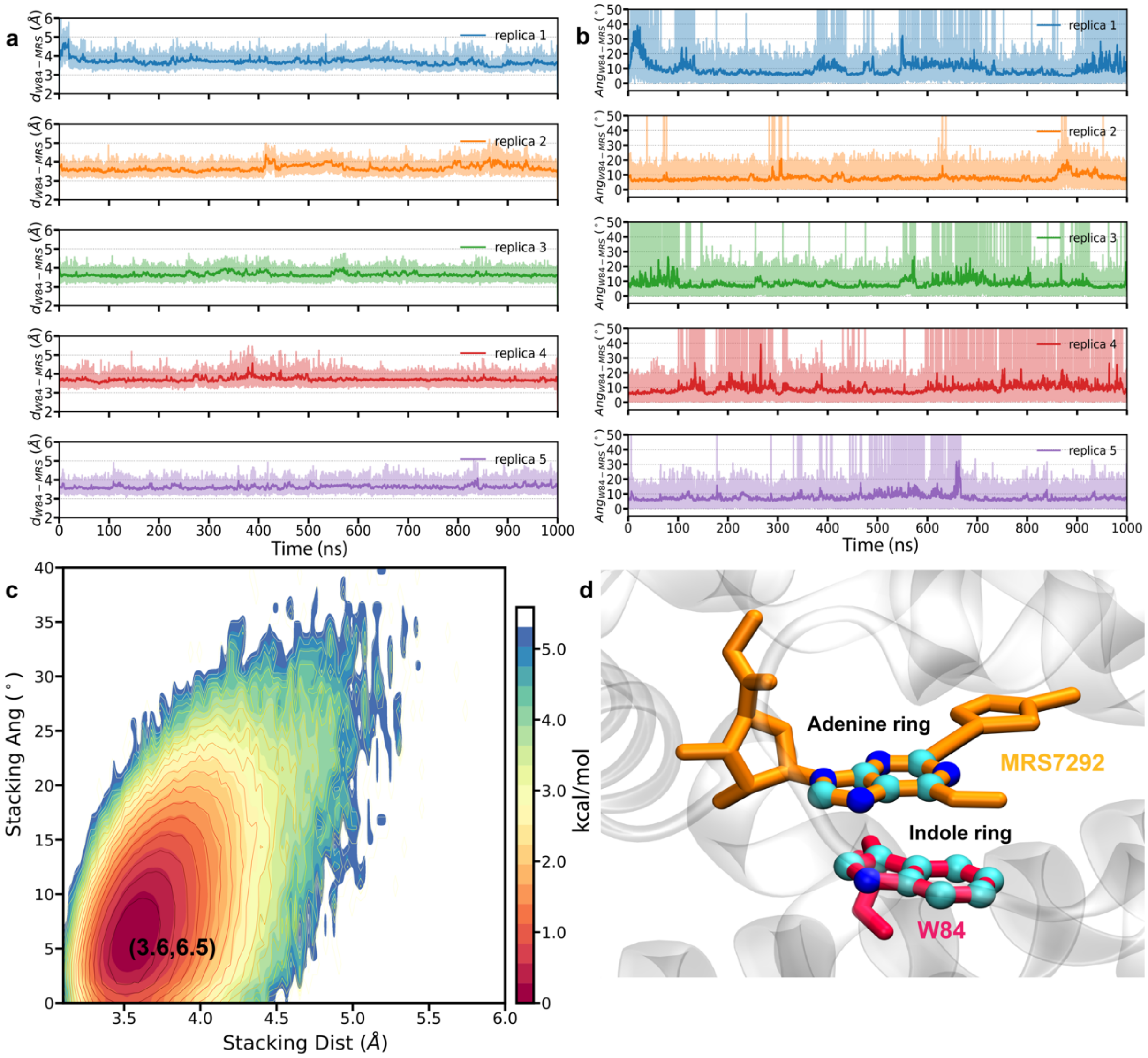

Supplementary Figure 8. π-π stacking of MRS7292 (adenine ring) and hDAT:W84 (indole ring). (a) COM distances between the adenine and indole ring atoms (shown in space-filling in (d)). (b) The angle between the normal vectors of the rings. (c) The free energy landscape of the π-π stacking between W84 and MRS7292 depicted in the distance-angle space. Free energies are derived from the 5 μs aggregate simulations. The stacking distances (x-axis) are between the COM of the adenine and indole rings, and the stacking angles (y-axis) are between the normal vectors of the rings. (d) Simulation snapshot highlighting the stacking interaction between MRS7292 (orange) and W84 (red).

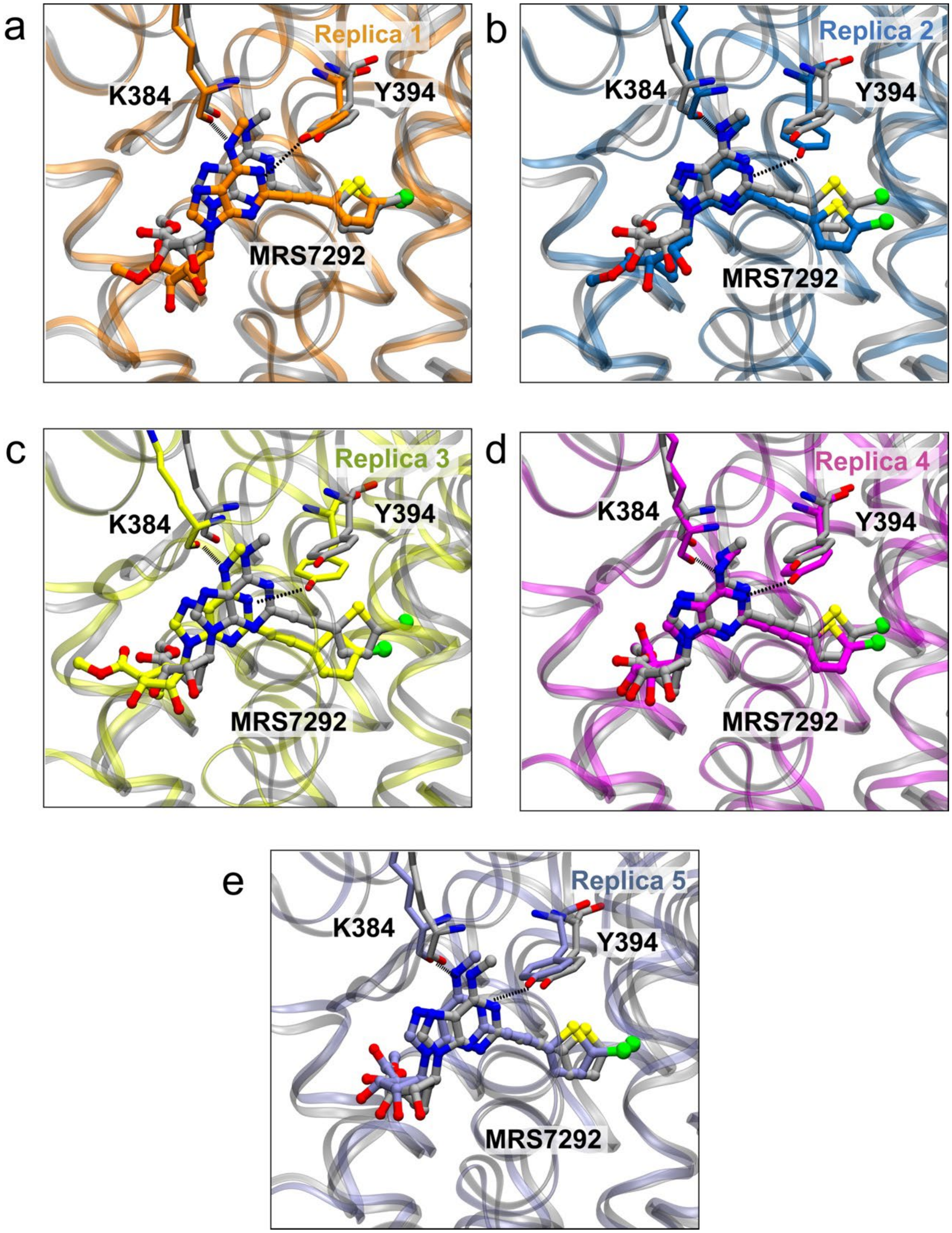

Supplementary Figure 9. **Comparing the cryo-EM ligand pose and those in the five simulation replicas for MRS7292.** (a-e) Superimposed, close-up view of the MRS-site and MRS7292 in the allosteric pocket. The cryo-EM model is shown in silver in each panel, and the final conformations of simulation replicas 1-5 are shown in orange, blue, light green, magenta, and lavender, respectively. Two important hydrogen bonds between Δ-hDAT:K384 and Y394 and MRS7292@N2 and N5 are shown with dashed lines. Δ-hDAT residues are depicted in stick representation.
